# An analysis of pharmacy workforce capacity in Nigeria

**DOI:** 10.1186/s40545-018-0147-9

**Published:** 2018-09-03

**Authors:** Aniekan Ekpenyong, Arit Udoh, Eneyi Kpokiri, Ian Bates

**Affiliations:** 1 University of Uyo, Uyo, Akwa Ibom State Nigeria; 2 Anglia Ruskin University, Chelmsford, UK; 3 University College London, London, UK

## Abstract

**Background:**

Pharmacists are critical for attaining the goal of universal health coverage and equitable access to essential health services, particularly in relation to access to medicines and medicines expertise. We describe an analysis of the pharmacy workforce in Nigeria from 2011 to 2016 in order to gain insight on capacity and to inform pharmacy workforce planning and policy development in the country.

**Method:**

The study was conducted using census data obtained from the Pharmacists Council of Nigeria (PCN) via a validated data collection tool. The statistical methods used for analysis were descriptive (frequencies, percentages, mean) and linear regression. Secondary data on population distribution per state was obtained from the Federal Bureau of Statistics and the National Population Commission (NPC) of Nigeria.

**Result:**

The data showed 21,892 registered pharmacists with only 59% (*n* = 12,807) in active professional practice. There are also more male (62%) compared to female pharmacists while 42% of the licensed workforce with known area of practice are in community practice followed by hospital pharmacy (11%). A rise in number of pharmacists (0.53–0.66) and new pharmacy graduates per year (0.062–0.083) per 10,000 population was observed over the five years analysed; however the overall density remains significantly low. Pharmacists’ density also varied considerably between states (Median = 0.39; Min - Max: 0.05–4.3). Regionally, more than a third (~ 40%) of the licensed workforce and community pharmacies are situated in the South West region with fewer than 10% of the total in the North East and North West regions combined. A steady decline in number of pharmacists requesting a “letter of good standing” from PCN, a proxy measure of intent to migrate was also observed.

**Conclusion:**

The data indicate ongoing deficits in availability and supply of pharmacists in the country with widespread variance in distribution observed across the 36 states and the Federal Capital Territory (FCT). The findings suggest that observed deficits are not solely related to out-migration and highlights the need for policies that will promote increased within-country availability, equitable distribution and retention, especially in the underserved regions of North East and North West of Nigeria.

**Electronic supplementary material:**

The online version of this article (10.1186/s40545-018-0147-9) contains supplementary material, which is available to authorized users.

## Background

Health workers are critical for attaining the goal of universal health coverage and equitable access to essential health services [1]. Existing data indicate a global shortage of 7.2 million health workers including pharmacists and this figure is expected to rise to 12.9 million by 2035 [1–3]. Health workforce shortages are most severe in mid and low income regions of the world with countries in Sub-Sahara Africa (SSA) showing one of lowest density of health workers globally, despite bearing about a quarter of the global burden for preventable diseases [1, 4]. Pharmacists— as the third largest and most accessible healthcare professionals in the world [5, 6] are often the first point of contact with the health system in many countries [7–9]. Pharmacists are central to attaining the goal of equitable access and rational use of medicines [10, 11] — a key objective of universal health coverage.

Global reports on the capacity of the health workforce have focused mainly on doctors, nurses, midwives, health support workers and also provide policy recommendations for improving availability and access to these cadres of health workers [1, 4]. Other reports by the International Pharmaceutical Federation (FIP) and the Royal Pharmaceutical Society (RPS) Global Pharmacy Workforce Observatory also provide country-level data on the pharmacy workforce [2, 10–13]; however, data on absolute capacity and distribution of pharmacists and other pharmacy support staff is lacking for most countries in SSA including Nigeria. This lack of reliable data makes it difficult to adequately plan and respond to the health workforce crisis in relation to the availability of medicines expertise and delivery of pharmaceutical care services. This study assesses the capacity of the pharmacy workforce in the Federal Capital Territory (FCT) and 36 states of Nigeria with the overall objective of obtaining evidence to inform pharmacy workforce planning and policy development in the country. This project forms part of a strand of research that aim to gain insight and obtain evidence to inform pharmacy workforce planning and policy development in mid- and low-income countries.

### Country profile

Nigeria is the most populous country in Africa and the seventh in the world with a population of approximately 193 million people [14]. It comprises the Federal Capital Territory (FCT, Abuja) and 36 states that are subdivided into 774 Local Government Areas (LGAs) [15]. The states and FCT are also further grouped into six geopolitical regions: North East, North West, North Central, South East, South West and South South regions. These groupings are based on culture, historical background and geography with the country’s educational, political and economic resources shared across the six regions [15]. Existing economic and health indicators class Nigeria in the low mid-income bracket with an estimated population growth rate of 2.6% per year [16]. About 46% of the population in the country live in urban areas with reports showing life expectancy of 53 and 56 years for males and females, respectively [16]. Infectious diseases such as malaria, HIV/AIDs and tuberculosis are a leading cause of death in the country [16]. Although current estimates indicate improvement in under-five and maternal mortality rate between 1990 and 2012 in Nigeria, these are still significantly high at 117/1000 and 560/100,000 live births, respectively, compared to other countries in the SSA region [16]. Evidence also indicate an increasing prevalence of non-communicable and chronic diseases including diabetes, hypertension, cardiovascular diseases and stroke in the country [17–21]. Overall health status and access to healthcare varies across the different regions in the country with existing reports suggesting that health indicators are generally worse in the northern region compared to the Southern [22].

### National Health System in Nigeria

The healthcare system in Nigeria is split broadly into the public and private sector [23]. The private sector comprises 38% of the healthcare facilities in the country and also provides 60% of orthodox health services [24]. The public health sector which is fully government owned is categorised into three levels of care: the primary, secondary and tertiary care levels; with provision of health services at each of these levels largely the responsibility of the local, state and federal tiers of government, respectively [24, 25]. Primary healthcare centres in the country provide essential and preventative health services and are mainly located in rural areas [26, 27]. Secondary and tertiary healthcare facilities are mainly situated in urban areas and serve as referral hospitals for complex and specialised healthcare requirements [23–25, 28]. In practice, fluidity exist in the provision of health services within these established levels of care with limited funding, under staffing, non-availability of essential medicines and perceived lower quality of health services in the primary care facilities making the tertiary healthcare service providers the preferred choice in the health seeking behaviour of the general populace [23, 25, 26]. Critical shortages and inequitable distribution of health workers have also been reported in Nigeria including within existing health facilities in the different levels of care in the country [22, 24, 25, 29]. Studies indicate only about 20% of physicians, 30% of nurses, and 38% of the pharmaceutical workforce including pharmacy support staff work within the primary care facilities [22]. Healthcare financing in the public sector is shared between the local, state and federal government and to supplement for government spending; user fees are also charged at the point of service provision, although these fees are relatively smaller compared to those charged by private for-profit health service providers [25]. Individual out-of-pocket payment for healthcare services is the main stay of health expenditure in the country and accounts for about 75% of total health spending [25]. A National Health Insurance Scheme (NHIS) in Nigeria was established in 1999 and implemented in 2003 as a social security system with the objective of providing affordable health for all through a prepayment system of regular token contributions by the scheme’s participants [30, 31]. However, reports show that access to health services under the scheme is limited [32] with studies indicating only a 4% coverage [33].

### Pharmacy in Nigeria

The pharmaceutical sector in Nigeria is made up of the academia, administrative, regulatory, community (retail), industry and hospital practice areas and is regulated by the Pharmacists Council of Nigeria (PCN) [34–36]. Administrative pharmacists in Nigeria work in a variety of administration roles in hospitals, health institutions and government organisations. The Council determines the standard of knowledge and skills of individuals seeking to become pharmacists, maintains the register of persons entitled to practice as pharmacists, inspects, approve and licenses pharmaceutical premises. PCN also organises mandatory continuing professional development (MPCD) for pharmacists in the country [35, 36], however, at present this is not an enforced criterion for ongoing licensure. There are 18 accredited pharmacy training institutions in the country [36], representing an increase from the 13 schools of pharmacy reported in 2014 [35]. Graduate pharmacy students are registered provisionally with full registration status achieved after the completion of a compulsory one year internship training programme [34]. Licensure examinations are not required for pharmacy graduates of accredited institutions in Nigeria. However, foreign trained graduates must pass a licensure examination and complete a one year internship prior to registration to practice as a pharmacist. All of the pharmacy training institutions in the country offer both undergraduate and post graduate training courses in pharmacy. The minimum degree required to become a pharmacist in Nigeria is the Bachelor of Pharmacy (BPharm) which was introduced in the 1980s by the PCN [35]. Currently, there are plans to make the Doctor of Pharmacy (PharmD), the minimum entry requirement to practice as a pharmacist in Nigeria, as seen in other parts of the world [34, 35]. However, only the University of Benin which is situated in Edo State is accredited to award the degree, although there are reports that suggest existing plans to accredit the other pharmacy institutions for this award [34].

## Methodology

Data for this study was provided by PCN from the Pharmacists’ register in Nigeria. A data collection tool validated in previous research [2, 13] and originally developed by the International Pharmaceutical Federation Education Initiative (FIPEd) was adapted and forwarded to PCN through the office of the Registrar in November 2016 with a further reminder in January 2017. Data on absolute number of pharmacists per state, pharmacists’ distribution per gender and sector as well as number of pharmacy premises, pharmacy training institutions, and requests for letters of good standing (a proxy measure of migration) was provided by PCN with the completed data collection tool returned via email in February 2017. Secondary data on the general population and distribution per state was obtained online from the Federal Bureau of Statistics and National Population Commission (NPC) of Nigeria [37].

### Data analysis

Data was cleaned, crosschecked and analysed using the Statistical Package for Social Sciences (SPSS) version 22. Descriptive analysis using frequencies and percentages was conducted to determine the capacity of the workforce with respect to gender distribution, area of practice and distribution of pharmacists and pharmacies per state and the FCT standardized by population. Linear regression was also conducted to determine the relationship between number of pharmacists’ and community pharmacy per state per 10,000 population. Overall pharmacy workforce supply capacity was assessed via an analysis of availability of training institutions and number of new graduates per year while number of pharmacists requesting “letter of good standing” from PCN was evaluated as a proxy measure of intention to migrate and exit the workforce.

## Results

There are 21,892 registered pharmacists in Nigeria, however, the data suggest that only 12,807 (58.5%) are in active professional practice as indicated by the number of licensed pharmacists in 2016. Data on number of registered pharmacists who had retired or exited the workforce was not available. Gender distribution (which was only available for year 2016) showed there are more male (62%) than female pharmacists in the country. A steady increase in the number of registered and licensed pharmacists as well as number of graduates per year was observed from 2011 to 2016, although overall pharmacists’ and pharmacy graduate density per 10,000 population in the country remains significantly low (Table 1).

**Table 1:** Number of Pharmacists and Pharmacy Graduates 2011–2016

Year	Country population (millions) ^α^	N Registered Pharmacists	N Licensed Pharmacists (%)	Density of Pharmacists* (per 10,000 population)	N New Graduates per year	Density of New Graduates per year (per 10,000 population)
2011	164.80	15,884	8746 (55)	0.53	1028	0.062
2012	170.16	16,779	9398 (56)	0.56	1040	0.061
2013	175.69	17,847	10,402 (58)	0.59	1274	0.072
2014	181.40	19,063	11,322 (59)	0.62	1460	0.081
2015	187.30	20,507	11,837 (58)	0.63	1548	0.083
2016	193.39	21,892	12,807 (59)	0.66	1596	0.083

*Density was calculated using the number of licensed pharmacists as this represents the active workforce and provides a more reliable estimate of current capacity

^α^Official 2016 estimate of population per state obtained from the Federal Bureau of Statistics and National Population Commission available at http://nigerianstat.gov.ng/elibrary

Data on distribution per area of pharmacy practice showed only 42% of the total licensed pharmacists are known to be in community practice, followed by hospital practice (11%). Of the 7465 licensed pharmacists with specified area of practice, majority were in community practice (72%) (Table 2). The data also indicated that 8.6% (*n* = 1100) of the licensed workforce were unemployed, 33% (*n* = 4175) were undergoing national youth service (NYSC), and 67 foreign trained pharmacists had no specified area of practice. Data on the distribution of pharmacists in private and public sector was not available.

**Table 2:** Distribution of pharmacists per area of practice

Area of Practice	N Licensed Pharmacists (% Total)	N licensed pharmacists with known area of practice (%)
Academia	106 (0.8)	106 (1.42)
Administrative & regulatory	331 (2.6)	331 (4.43)
Industry	227 (1.8)	227 (3.04)
Hospital	1421 [11]	1421 [19]
Community	5380 (42)	5380 (72)
Others*	5342 (41.7)	–
Total	12,807	7465

*includes 67 licensed pharmacists with unspecified area of practice, 1100 licensed but unemployed pharmacists, and 4175 pharmacists undergoing national youth service (NYSC). NYSC is a one-year mandatory service for recent graduates and involves working in government institutions and not necessarily as a pharmacist

**Fig. 1:**
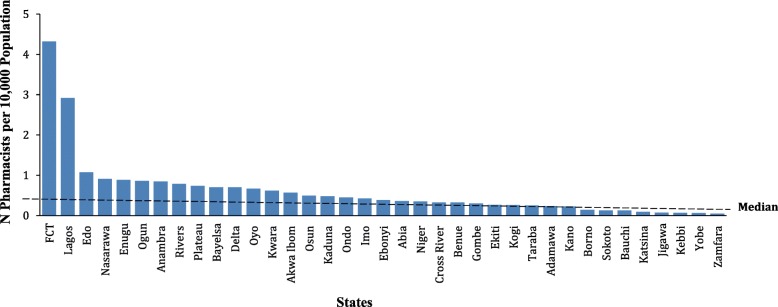
Number of pharmacists per state per 10,000 population

Majority of the licensed workforce in the country are in Lagos State (*n* = 3661, 29%) and the FCT (*n* = 1540, 12%) while Yobe and Zamfara States had the least (*n* = 20 and 22, respectively) (Fig. 1). Pharmacists density (per 10,000 population and available for year 2016 only) also varied significantly between states and ranged from 0.05 in Zamfara to 4.3 in the FCT (median density = 0.39). In addition, there was minimal correlation between number of pharmacists and the population per state and this was less than 20% variance overall (R^2^ = 0.19, *p* < 0.001) and 10% variance without the extreme values from Lagos State and the FCT (R^2^ = 0.10, *P* < 0.001). Gender distribution was consistent across the states with the highest proportion of males in Kebbi State (86%) and the least in the FCT (54%) (Full Data Table presented in Additional file 1).

**Fig. 2:**
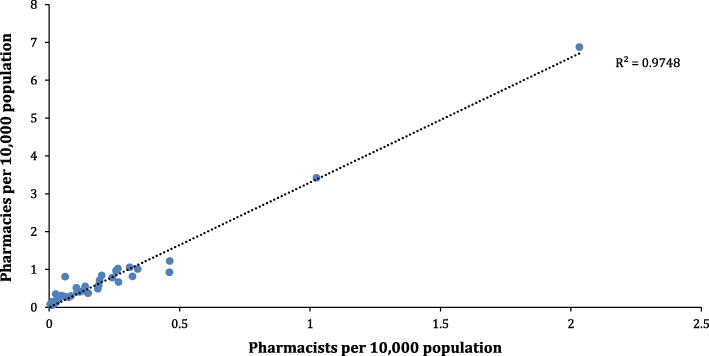
Number of pharmacists and community pharmacies per state per 10,000 population

There are a total of 3768 registered community pharmacies in the country with a disproportionately higher number situated in Lagos State (*N* = 1096; 29%) and the FCT (*N* = 455; 12%) compared to Jigawa, Yobe and Zamfara with fewer than 5 each (median = 50; Min-Max: 2–1096). Overall, approximately half of the states in the country had fewer than 50 pharmacies each (Full Data Table presented in Additional file 1). There was a strong correlation between number of community pharmacies and pharmacists per 10,000 population (R^2^ = 0.97; Fig. 2) indicating that states with higher number of pharmacists had a greater tendency to have more community pharmacies. States in the South West region had the highest number of licensed pharmacists and community pharmacies (although this was mainly within Lagos state) while the North East region had the least (Table 3). Regional distribution of pharmacists per area of practice was also consistent with the national distribution with community practice showing the highest number of licensed pharmacists with specified area of practice followed by hospital practice (Table 3). The data also showed 18 institutions accredited to offer undergraduate and postgraduate pharmacy training in the country and these are distributed across sixteen states with two institutions per state in Edo and Rivers and one each in fourteen states (Data Table 1 in Additional file 1). Regionally, these institutions are disproportionately distributed across the six geopolitical regions with the highest number observed in the South South region (*N* = 7; Table 3). Data on the number of registered pharmacies situated in hospitals was not available.

**Table 3:** Regional distribution of licensed pharmacists, community pharmacies and pharmacy training institutions in Nigeria

Region	Population (millions)*	N Licensed Pharmacists (% Total)^β^	Gender (%)		N Pharmacists by area of practice (%)						N Community Pharmacies (% Total)	N Pharmacy Training Institutions
			Male	Female	Acad & Reg.	Admin	Hosp	Comm	Indus	Other ^α^		
North East	26.26	456 (3.6)	73	27	0.4	6.1	20.6	38.2	0.7	34.0	72 (1.9)	1
North West	48.92	916 (7.2)	73	27	1.6	3.3	14.4	42.4	1.3	37.0	179 (4.7)	2
South East	21.96	1328 (10.5)	61	39	1.0	2.6	11.1	45.9	1.8	37.6	377 (10)	2
South South	28.83	2018 [16]	60	40	1.2	1.6	12.7	36.9	1.0	46.6	794 (21.1)	7
North Central	29.25	2771 (21.9)	60	40	0.7	3.6	13.2	39.9	0.8	41.8	809 (21.5)	2
South West	38.26	5158 (41)	62	38	0.6	2.0	8.1	44.7	2.8	41.9	1537 (40.8)	4

*population per region/states used are the 2016 estimates published by the Federal Bureau of Statistics of Nigeria available at http://nigerianstat.gov.ng/elibrary

^α^includes licensed pharmacists undergoing NYSC and others with unspecified areas of practice who are unemployed and/or foreign trained new graduates

^β^excludes 160 pharmacists not residing in the country and whose licenses are not accounted for within the states/regions

*Acad* Academic, *Admin & Reg* Administrative & regulatory, *Hosp* Hospital, *Comm*: Community, *Indus* Industrial area of practice, respectively

**Fig. 3:**
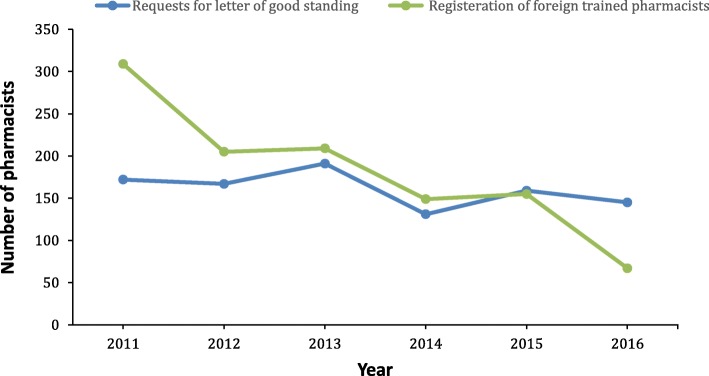
Number of requests for letters of good standing and foreign trained pharmacists

In total, 2825 (22%) pharmacists participated in the mandatory continuing professional development programme (MCPD) for the year 2016, representing less than one-fourth of the licensed workforce (data on attendance for 2011 to 2015 was not available). A decline in number of pharmacists requesting “letter of good standing” from PCN (a proxy measure of intent to migrate) was observed between 2011 and 2016 (Fig. 3). A similar trend was also observed in the number of foreign trained pharmacists registering with the PCN.

## Discussion

Evidence from this study indicate that most of the licensed pharmacists with known area of practice in Nigeria are in community and hospital practice. While the finding is comparable to the overall global trend [10]; the limited number of pharmacists in academic, industry and administrative pharmacy demonstrate significant deficits in these key areas of practice in the country. Policies that would facilitate recruitment of pharmacists to these areas are therefore needed urgently, so as to ensure increased availability of pharmaceutical expertise for the drug manufacturing and human resources for pharmacy in the country. The study results also suggests that up to 40% of the workforce are not actively involved in pharmacy practice, in a finding that is similar to that of a published report from Ghana [38] as well as the trend in the medical profession in Nigeria [24]. Registered pharmacists who practice in academia, industry and administrative pharmacy in Nigeria may not renew their licenses primarily because there is no strict enforced requirement for annual renewal in these areas practice. This feature however does not fully account for the disparity between number of registered and licensed pharmacists observed; particularly in view of existing evidence from global and country level trend reports that show that pharmacists in industrial, academic and administrative practice comprise less than 10% of the overall pharmacy workforce [2, 10, 38–41]. Further studies that would provide insight are therefore necessary, although similar studies from other countries in Africa suggest this may be associated with low job satisfaction due to perceived lack of opportunities for career advancement and limited incentives including remuneration in pharmacy [38–40, 42].

The increase in pharmacists density observed over the five years analysed is likely linked with the rise in number of training institutions and pharmacy graduates per year in the country. Despite the observed trend increase, pharmacists density is still significantly low at less than one pharmacist per 10,000 population compared to density in other countries such as Jordan (20.87), Canada (10.17), USA (8.82) and United Kingdom (8.08) [11]. This indicates continued pharmacy workforce shortages and as a corollary, challenges with access to medicines and pharmaceutical expertise in the country. The disproportionate and inequitable distribution of pharmacists and pharmacies across the states and regions is similar to evidence from published research in other African nations including Tanzania [42], Ethiopia [40], Sudan [39] and Ghana [38] that demonstrate larger proportion of pharmacists in cosmopolitan areas. The finding is also similar to the trend within the medical profession in Nigeria [24] and highlights the need for strategies that would facilitate recruitment and retention of pharmacists in the underserved areas, particularly those within the North East and North West regions of the country.

The overall gender composition of the pharmacist workforce in Nigeria is in contrast to existing reports that indicate a rising trend increase in number female pharmacists globally [2, 10]. Our finding however is consistent with evidence from reports that demonstrate sample means of 68% for male pharmacists in countries within the African region [10]. Furthermore, the increase in number of graduates per year with corresponding trend increase in graduate density per 10,000 population observed over the five years analysed is similar to evidence from other African countries [43]. However, the overall density of pharmacy graduates in Nigeria still remains significantly low, indicating continuing deficits in pharmacy human resources supply capacity compared to higher income countries such as those in Europe that show mean sample density of 0.41 [43]. The finding of this study, which is in line with other published research [43] also suggest that establishing more pharmacy schools particularly in underserved regions of the country and expanding the current capacity of existing schools would likely facilitate a corresponding increase in the pharmacy workforce population. This explains the continued shortages in availability of pharmacists in Nigeria given that pharmacy training institutions (defined as institutions providing undergraduate and post-graduate pharmacy training) are vital for sustaining and maintaining the supply of well-trained pharmacists [43]. Although our data showed pharmacists’ unemployment rate of 8.6%, it was not clear whether these pharmacists are employed in areas unrelated to pharmacy, as this information was not available. Our finding does suggest that more has to be done to absorb available pharmaceutical expertise into the workforce, particularly in sectors where there are significant deficits such as industrial and academic pharmacy.

The finding that more than a third of the licensed pharmacists in 2016 are undergoing National Youth Service Corps (NYSC) suggests that a significant proportion of the active workforce are new entrants. NYSC is a one-year mandatory service for recent graduates and involves working in government institutions with minimum pay [44]. The finding corroborates existing report that show that recent graduates account for a large number of new recruits in public sector health facilities in Nigeria [22]. Effective utilisation of pharmacists under the NYSC scheme in underserved regions of the country may be short term strategies that can improve the availability of pharmacists in these areas. The Australian rural pharmacy workforce programme is a notable example of an initiative aimed at recruiting, retaining and retraining the pharmacy workforce and involve strategies such as provision of undergraduate and postgraduate scholarships, emergency locum scheme, rural intern training and incentive allowance as well as rural pharmacy maintenance allowances **f**or pharmacist in underserved areas [45]**.**

The finding that only 22% of the workforce took part in MCPD programme in 2016 suggests the need for measures that will increase uptake. Follow up data on reasons for the low interest levels was not available, however, this may not be unrelated to evidence from studies in other countries that indicate that lack of knowledge about MCPD, poor access to the internet, time constraints and high monetary cost contribute to low uptake [46, 47]. Our finding is similar to that from studies in Scotland and USA that also show that there is a general lack of motivation and low level of knowledge of pharmacists on the benefits of continuing development programmes [48, 49]. Increased sensitization and timely provision of MCPD related information may increase uptake in Nigeria.

The results of this study showed a decline in out-migration of pharmacists abroad as evident in the steady decrease in number of letters of good standing requested over the five years assessed. Individual “letters of good standing” are provided by the PCN to licensed pharmacists on request and are essential for the registration of foreign trained and/or licensed pharmacists in many countries. The letters therefore serve as a proxy indicator for migration, and even though it does not provide complete information on rate of migration from the pharmacy workforce, it does suggest a decline in migration of pharmacists from the country. This finding is corroborated by evidence from medical and nursing profession in Nigeria [24] and from pharmacy in Sudan [39] in contrast to existing evidence that suggests the rate of out-migration and brain drain of health workers in countries in Africa has accelerated over the years [50]. Our study finding also suggests that migration alone is not the critical reason for observed deficits and shortages in the pharmacy workforce in Nigeria. Further studies on workforce mobility between states, regions and pharmacy sectors as well as from rural to urban areas are also crucial in order to provide critical insights necessary for workforce planning and projections.

### Study scope and limitation

This study relied on census data provided by the PCN as well as secondary data on the population distribution per state from online databank of the Federal bureau of Statistics and the National Population Commission of Nigeria. The general population data used in the analysis were the 2016 estimates of the Federal Bureau of Statistics of Nigeria and the National Population Commission of Nigeria [37]. These estimates are not exact, particularly because the last census carried out in the country was in 2006 and a population count is planned for 2018. Therefore it is probable that actual pharmacy workforce capacity indicators are likely to be relatively lower than those in this study [51]. Challenges with missing and incomplete data from PCN are also a key source of bias in this study. For example, missing data on gender distribution and workforce composition per state per year limited our ability to compare trend improvement in the states and FCT. Furthermore, it was not clear whether the discrepancy between number of registered and licensed pharmacists was due only to a proportion of the workforce not being in active professional practice. Data on exit from the workforce due to death, retirement and other reasons could have provided additional insight on this issue, however, these were not available. This study did not attempt to map the practice profile of pharmacists in the country and as a result, it was not possible to comment on pharmacists’ distribution in the private and public sectors. Also, this report focused on the pharmacists as data on distribution of pharmacy support staff such as technicians were not available, although the PCN oversees the training and regulation of this cadre of workers. Availability of data on support staff would have provided a broader outlook on the overall composition of the pharmaceutical workforce in the country.

## Conclusion

These study findings indicate ongoing deficits in availability of pharmacists in Nigeria with widespread variance in distribution observed across the 36 states and FCT. These deficits are not related to out-migration and the inequitable distribution also observed add to the complexity in effective workforce planning. Our findings highlight the need for policy mechanisms and strategic solutions that will promote increased availability, equitable distribution and retention of pharmacists, especially within the underserved regions of the North East and North West of the country. These policies are needed urgently to ensure equitable access to pharmaceutical expertise in the FCT and 36 states of the country.

## Additional file

Additional file 1: Data set included as supplementary material (Appendix 1). (DOCX 38 kb)

## Abbreviations

BPharm: Bachelor of Pharmacy
DPharm: Doctor of Pharmacy
FCT: Federal Capital Territory
FIP: International Pharmaceutical Federation
HIV/AIDS: Human immunodeficiency virus and acquired immune deficiency Syndrome
LGA: Local Government Area
MCPD: Mandatory continuing professional development
NHIS: National Health Insurance Scheme
NPC: National Population Commission of Nigeria
NYSC: National Youth Service Corps
PCN: Pharmacists Council of Nigeria
RPS: Royal Pharmaceutical Society
SPSS v22: Statistical package for the social sciences version 22
SSA: Sub-Sahara Africa
YPG: Young Pharmacists Group
