# Supplementary material for: An analysis of pharmacy workforce capacity in Nigeria
Source: J Pharm Policy Pract. 2018 Sep 3;11:20. doi: 10.1186/s40545-018-0147-9 (PMC6120071; doi:10.1186/s40545-018-0147-9)
Supplement: Supplementary file 1 — Data set included as supplementary material (Appendix 1). (DOCX 38 kb) [file 40545_2018_147_MOESM1_ESM.docx]

| **APPENDIX 1**  **Data Table 1** | | | | | | | | | | | | | | | | |
| --- | --- | --- | --- | --- | --- | --- | --- | --- | --- | --- | --- | --- | --- | --- | --- | --- |
| **Geopolitical Regions/State** | **N Pharmacists** | | | | | | **Unemployed** | **Foreign trained pharmacists with sector unspecified** | **N Total** | **Male (N)** | | **Female (N)** | | **Retail pharmacies** | | **Number of pharmacy training institutions** |
|  | **Comm.** | **Hosp.** | **Indus.** | **Acad.** | **Admin.** | **National service (NYSC)** |  |  |  |  |  |  |  |  |  |  |
| **North East** | | | | | | | | | | | | | | | | |
| Adamawa | 38 | 12 | 2 | 0 | 10 | 34 | 3 | 0 | 99 | | 77 | 22 | 27 | | 0 | |
| Bauchi | 27 | 22 | 0 | 1 | 4 | 25 | 3 | 0 | 82 | | 46 | 36 | 7 | | 0 | |
| Borno | 34 | 15 | 1 | 0 | 1 | 26 | 4 | 0 | 81 | | 57 | 24 | 12 | | 1 | |
| Gombe | 37 | 23 | 0 | 1 | 5 | 27 | 5 | 0 | 98 | | 72 | 26 | 7 | | 0 | |
| Taraba | 29 | 16 | 0 | 0 | 8 | 21 | 2 | 0 | 76 | | 63 | 13 | 16 | | 0 | |
| Yobe | 9 | 6 | 0 | 0 | 0 | 4 | 1 | 0 | 20 | | 17 | 3 | 3 | | 0 | |
| **North West** | | | | | | | | | | | | | | | | |
| Jigawa | 19 | 11 | 0 | 0 | 2 | 7 | 3 | 0 | 42 | | 36 | 6 | 2 | | 0 | |
| Kaduna | 175 | 47 | 3 | 13 | 11 | 117 | 29 | 0 | 395 | | 254 | 141 | 98 | | 1 | |
| Kano | 117 | 40 | 8 | 0 | 4 | 102 | 21 | 0 | 292 | | 241 | 51 | 43 | | 0 | |
| Katsina | 26 | 18 | 1 | 1 | 7 | 16 | 4 | 0 | 73 | | 48 | 25 | 14 | | 0 | |
| Kebbi | 21 | 3 | 0 | 0 | 2 | 2 | 1 | 0 | 29 | | 25 | 4 | 6 | | 0 | |
| Sokoto | 20 | 11 | 0 | 1 | 2 | 28 | 1 | 0 | 63 | | 52 | 11 | 12 | | 1 | |
| Zamfara | 10 | 2 | 0 | 0 | 2 | 6 | 2 | 0 | 22 | | 16 | 6 | 4 | | 0 | |
| **North Central** | | | | | | | | | | | | | | | | |
| Benue | 76 | 32 | 0 | 1 | 3 | 63 | 11 | 0 | 186 | | 138 | 48 | 73 | | 0 | |
| Kogi | 53 | 25 | 1 | 0 | 4 | 29 | 4 | 0 | 116 | | 90 | 26 | 32 | | 0 | |
| Kwara | 91 | 24 | 2 | 3 | 7 | 52 | 17 | 0 | 196 | | 127 | 69 | 53 | | 1 | |
| Nasarawa | 85 | 30 | 0 | 0 | 2 | 83 | 29 | 1 | 230 | | 145 | 85 | 67 | | 0 | |
| Niger | 122 | 20 | 1 | 0 | 2 | 40 | 8 | 0 | 193 | | 138 | 55 | 55 | | 0 | |
| Plateau | 121 | 44 | 2 | 8 | 5 | 104 | 26 | 0 | 310 | | 204 | 106 | 74 | | 1 | |
| FCT | 558 | 192 | 15 | 7 | 77 | 533 | 151 | 7 | 1540 | | 837 | 703 | 455 | | 0 | |

| **Licensed pharmacists not residing in the country.* |
| --- |

| **Geopolitical Regions** | **Comm.** | **Hosp.** | **Indus.** | **Acad.** | **Admin.** | **National service (NYSC)** | **Unemployed** | **Foreign trained pharmacists with sector unspecified** | **N Total** | **Male** | **Female** | **Retail pharmacies** | **Number of pharmacy training institutions** |
| --- | --- | --- | --- | --- | --- | --- | --- | --- | --- | --- | --- | --- | --- |
| **South East** | | | | | | | | | | | | | |
| Abia | 79 | 10 | 3 | 0 | 9 | 24 | 10 | 0 | 135 | 87 | 48 | 35 | 0 |
| Anambra | 274 | 38 | 9 | 6 | 8 | 104 | 27 | 0 | 466 | 276 | 190 | 123 | 1 |
| Ebonyi | 33 | 14 | 0 | 0 | 4 | 46 | 13 | 1 | 111 | 69 | 42 | 33 | 0 |
| Enugu | 134 | 61 | 7 | 5 | 9 | 118 | 53 | 2 | 389 | 227 | 162 | 100 | 1 |
| Imo | 90 | 24 | 5 | 2 | 5 | 77 | 23 | 1 | 227 | 135 | 92 | 86 | 0 |
| **South South** | | | | | | | | | | | | | |
| Akwa Ibom | 130 | 47 | 0 | 4 | 1 | 100 | 28 | 0 | 310 | 197 | 113 | 123 | 1 |
| Cross River | 59 | 17 | 2 | 0 | 3 | 35 | 10 | 0 | 126 | 101 | 25 | 50 | 0 |
| Bayelsa | 33 | 26 | 2 | 2 | 4 | 77 | 16 | 0 | 160 | 98 | 62 | 12 | 1 |
| Rivers | 203 | 60 | 5 | 3 | 11 | 220 | 70 | 0 | 572 | 312 | 260 | 284 | 2 |
| Delta | 163 | 60 | 5 | 2 | 8 | 118 | 40 | 0 | 396 | 235 | 161 | 154 | 1 |
| Edo | 156 | 46 | 7 | 13 | 5 | 188 | 39 | 0 | 454 | 261 | 193 | 171 | 2 |
| **South West** | | | | | | | | | | | | | |
| Ekiti | 36 | 14 | 1 | 0 | 4 | 25 | 6 | 1 | 87 | 57 | 30 | 13 | 0 |
| Lagos | 1671 | 235 | 109 | 18 | 76 | 1227 | 299 | 26 | 3661 | 2238 | 1423 | 1096 | 1 |
| Ogun | 191 | 42 | 16 | 0 | 7 | 149 | 42 | 1 | 448 | 276 | 172 | 150 | 1 |
| Ondo | 85 | 44 | 1 | 0 | 2 | 61 | 16 | 0 | 209 | 154 | 55 | 42 | 0 |
| Osun | 92 | 27 | 1 | 9 | 5 | 82 | 17 | 0 | 233 | 154 | 79 | 76 | 1 |
| Oyo | 230 | 54 | 14 | 5 | 9 | 165 | 41 | 2 | 520 | 306 | 214 | 160 | 1 |
| Other* | 53 | 9 | 4 | 1 | 3 | 40 | 25 | 25 | 160 | 96 | 64 | - | 0 |
| **Licensed pharmacists not residing in the country* | | | | | | | | | | | | | |
|  | | | | | | | | | | | | | |

**Data Table 1 *contd***

| **Data Table 2: Pharmacists and pharmacies per state per 10,000 population** | | | |
| --- | --- | --- | --- |
| **States** | **Population**  **(2016 estimates)^α^** | **Density of Pharmacists**  **(per 10,000 population)** | **Density of pharmacies**  **(per 10,000 population)** |
| Abia | 3727346 | 0.36 | 0.09 |
| Adamawa | 4248436 | 0.23 | 0.06 |
| Akwa Ibom | 5482177 | 0.57 | 0.22 |
| Anambra | 5527808 | 0.84 | 0.22 |
| Bauchi | 6537313 | 0.13 | 0.01 |
| Bayelsa | 2277960 | 0.70 | 0.05 |
| Benue | 5741814 | 0.32 | 0.13 |
| Borno | 5860182 | 0.14 | 0.02 |
| Cross River | 3866268 | 0.33 | 0.13 |
| Delta | 5663362 | 0.70 | 0.27 |
| Ebonyi | 2880383 | 0.39 | 0.11 |
| Edo | 4235594 | 1.07 | 0.40 |
| Ekiti | 3270798 | 0.27 | 0.04 |
| Enugu | 4411118 | 0.88 | 0.23 |
| FCT | 3564125 | 4.32 | 1.28 |
| Gombe | 3256962 | 0.30 | 0.02 |
| Imo | 5408756 | 0.42 | 0.16 |
| Jigawa | 5828162 | 0.07 | 0.00 |
| Kaduna | 8252365 | 0.48 | 0.12 |
| Kano | 13076891 | 0.22 | 0.03 |
| Katsina | 7831319 | 0.09 | 0.02 |
| Kebbi | 4440049 | 0.07 | 0.01 |
| Kogi | 4473490 | 0.26 | 0.07 |
| Kwara | 3192892 | 0.61 | 0.17 |
| Lagos | 12550598 | 2.92 | 0.87 |
| Nasarawa | 2523395 | 0.91 | 0.27 |
| Niger | 5556247 | 0.35 | 0.10 |
| Ogun | 5217716 | 0.86 | 0.29 |
| Ondo | 4671695 | 0.45 | 0.09 |
| Osun | 4705589 | 0.50 | 0.16 |
| Oyo | 7840863 | 0.66 | 0.20 |
| Plateau | 4200441 | 0.74 | 0.18 |
| Rivers | 7303923 | 0.78 | 0.39 |
| Sokoto | 4998089 | 0.13 | 0.02 |
| Taraba | 3066833 | 0.25 | 0.05 |
| Yobe | 3294136 | 0.06 | 0.01 |
| Zamfara | 4515427 | 0.05 | 0.01 |

|  |
| --- |
|  |

**^α^** official 2016 estimate of total population obtained from the National Population Commission Forecasts available at: <http://nigerianstat.gov.ng/elibrary>
